# C-Peptide and cardiovascular risk factors among young adults in a southern Brazilian cohort

**DOI:** 10.1186/s12902-018-0308-5

**Published:** 2018-11-06

**Authors:** Romildo Luiz Monteiro Andrade, Denise P. Gigante, Isabel Oliveira de Oliveira, Bernardo Lessa Horta

**Affiliations:** 1 University Hospital Cassiano Antônio de Moraes (HUCAM) of the Federal, University of Espírito Santo (UFES), Vitória-ES, Brazil; 2 Post-Graduate Program in Epidemiology, Federal University of Pelotas (UFPel), Pelotas-RS, Brazil; 3 Vitória, Brazil

**Keywords:** C-Peptide, Cardiovascular disease, Risk, Young adults

## Abstract

**Background:**

Proinsulin connecting peptide (C-Peptide) is a marker of the beta-cell function and has been considered a marker of insulin resistance whose evidence suggests were associated with cardiovascular mortality. Our study aims to evaluate the association of C-Peptide with metabolic cardiovascular risk factors among young adults followed since birth in southern Brazil.

**Methods:**

In 1982, maternity hospital in Pelotas, a southern Brazilian city, were visited daily and all births were identified. Live births whose family lived in the urban area of the city were identified, their mothers interviewed, and these subjects have been prospectively followed. Casual hyperglycemia patients were excluded from analysis. C-Peptide was assessed at 23 years, when transversely analyzed its association with cardiometabolic and hemodynamic risk factors, and longitudinally 30 years of age.

**Results:**

At age 23, 4297 individuals were evaluated, and C-Peptide was measured in 3.807. In a cross-sectional analysis at 23 years of age, C-Peptide was positively associated with waist circumference, body mass index, glycaemia, triglycerides, and C-reactive protein. The association with HDL cholesterol was negative. In the longitudinal analysis at 30 years, C-Peptide remained associated with BMI, waist circumference, glycated hemoglobin, triglycerides, and C-reactive protein, whereas the association was negative for HDL.

**Conclusion:**

In the Pelotas birth cohort, the C-Peptide was associated with obesity indicators (waist circumference and BMI) cross-sectional (23 years) and longitudinal (30 years). We also observed cross-sectional and longitudinal associations of C-Peptide with cardiometabolic and inflammatory risk factors**.**

## Background

In 2012, cardiovascular diseases were the main cause of death, accounting for 17.5 million deaths [1]. And three quarters of these deaths, occurred in low and medium-income countries (70% of the global population) [2]. In face of population ageing and the increase in the prevalence of diabetes and obesity, cardiovascular mortality is estimated to rise over the next decades [3, 4].

Proinsulin connecting peptide-(C-Peptide) is considered a marker of insulin resistance, epidemiological studies suggest its performance as a cardiovascular risk factor [5–7]. Secreted by β cells in the pancreas at amounts equimolar to insulin [8], C-Peptide is also a marker of beta cell function, and studies have shown its association to overall cardiovascular risk and mortality [9]. Its pathophysiological activity would be linked to the stimulation of vascular permeability to monocytes, stimulating the differentiation in macrophages, promoting the phagocytosis of molecules of oxidized lipoproteins, such as low-density lipoprotein (LDL), and differentiating into foam cells, the classic cellular substrate of atherosclerotic lesions [10]. C-Peptide would also act in subsequent phases of the atherogenic process, inducing the proliferation of smooth muscle cells and the cascade of release of pro-atherogenic components such as cytokines, metalloproteinases, and oxidative molecules, besides clotting factors such as the tissue plasminogen activator (tPA) [11, 12].

Population studies are in line with findings such as Cabrera et al. observed that individuals in the upper tercile of C-Peptide were at higher risk of coronary artery disease (RR 2.4; CI 95% 1.3–4.6) and myocardial infarction (RR 2.8; C I95% 1.1–6.9) [13]. Marx et al. reported that serum levels of C-Peptide in patients subjected to coronary angiography were associated with overall (HR 1.46 CI95% 1.15–1.85) and cardiovascular (HR 1.58 CI 95% 1.15–2.18) mortality [14]. Patel et al. evaluated individuals with fasting glycaemia ≥70 mg/dL and found that C-Peptide was associated with cardiovascular (HR 1.60, CI95% 1.07–2.39) and overall (HR 1.72, CI95% 1.34–2.21) mortality [15]. Min et al. also found an association with cardiovascular (HR 3.20 CI95% 2.07–4.93) and overall (HR 1.80 CI95% 1.33–2.43) mortality.

On the other hand, Bo et al. found no association between C-Peptide and cardiovascular mortality after adjusting for age, sex, body mass index, smoking, time on insulin therapy, glycated hemoglobin, systolic blood pressure, HDL cholesterol, triglycerides, and previous vascular complications [16]. However, the control for other metabolic cardiovascular risk factors may have blocked causal pathways between C-Peptide and mortality, underestimating the association.

Given the small number of studies assessing the association of C-Peptide with metabolic cardiovascular risk factors, and the possibility of analyzing their relation to the long period of follow-up we considered important to investigate such association. The present study aimed to evaluate the cardio-metabolic risk profiles in non-diabetic young stratified according to C-peptide quartiles in a southern Brazilian cohort [7].

## Methods

In 1982, the maternity hospital in Pelotas, a southern Brazilian city, were visited daily and all births were identified. The neonates whose families lived in the urban area were examined and the mothers interviewed soon after birth (n 5914). These individuals have been prospectively followed at different moments of their life cycles.

In 2004, an attempt was made to locate all participants of the cohort, using multiple strategies. The subjects were interviewed at home and invited to visit the research laboratory to donate a blood sample [17]. At 30 ages, a new attempt was made to locate all cohort members, who were invited to visit the research clinic, where they were interviewed, examined, and blood samples were collected [18].

Anthropometric variables of weight, was measured at 23 years, using Seca (Uniscale®, Germany) portable electronic scales with 100 g precision. Aluminum anthropometers with 1 mm precision were used to measure height according to Lohman et al. [19]. At 30 years, weight was measured with a scale connected to Bod Pod (Cosmed®, USA) device and height with an aluminum vertical stadiometer with 1 mm precision. Waist circumference was measured while the subjects were standing upright with feet together and arms relaxed along the body. The measuring tape was extended on the horizontal plane at waist height (narrowest portion of the trunk) between the last rib and the iliac crest at the end of a regular exhaling and without compressing the skin, and the value was recorded with 0.1 cm precision. Body mass index (BMI) was calculated by dividing the weight in kilograms (kg) by the square of the height in meters (m).

Cardiometabolic variables were measured at 23 years: capillary glycaemia (mg/dL), total cholesterol (mg/dL), HDL cholesterol (mg/dL), triglycerides (mg/dL), C-reactive protein (mg/dL). At age 30, serum glycaemia (mg/dL), total cholesterol (mg/dL); HDL cholesterol (mg/dL); LDL cholesterol (mg/dL), triglycerides (mg/dL); C-reactive protein (mg/dL); glycated hemoglobin (%) were measured. At 23 years, glycaemia was measured using an Accu-Chek Advantage (Roche®) portable glucometer, total cholesterol, HDL, and triglycerides were measured using a Selectra 2 (Merck®, Darmstadt, Germany. C-Peptide was measured in the blood samples collected at 23 years, using the chemiluminescence technique (Immulite – Siemens) [20], because the analyses were performed on samples collected at random, the estimates were adjusted for fasting time.

At 30 years, serum glucose was measured using the colorimetric enzyme assay with K082 -Glicose Monoreagente kits (Bioclin®, Brazil), LDL cholesterol was measured with K088 (Bioclin®, Brazil) kits and glycated hemoglobin (HbA1c), with K09 -HbA1c Bi-reagent (Bio-Rad®, Berkeley, California, USA) kits. High-sensitivity C-reactive protein (Hs-CRP) was measured using the automated turbidimetry technique with a BS 380 (Shenzhen-Mindray Bio-Medical Electronics Co.; Ltd®, China) chemical analyzer. Those measures with values below the lower limit of detection of 0.1 mg/L were converted to 0.05 mg/L. Expressing acute inflammatory conditions, we excluded values above 10 mg / L [21]. We also excluded those subjects whose serum glucose level was > 200 mg / dL at 23 and 30 years due to diabetes mellitus [22].

Blood pressure was measured at 23 years, using an Omron® wrist monitor at the beginning and at the end of the interview, about 60 min apart, with the respondent sited with the arm on a support. At 30 years, blood pressure was measured twice with an HEM705 CPINT (Omron®) automated device coupled to an arm cuff, which was replaced by a proper model for obese subjects. The mean of two measurements was used in the analyses.

The following variables were considered as confounding factors: physical activity was measured at 23 and 30 years of age using the International Physical Activity Questionnaire (IPAQ) [23] maternal skin color (assessed by the interviewer in the perinatal study), monthly family income in minimum wage, tobacco use (those subjects who reported smoking at least one cigarette a day at 23 years were considered as smokers), alcohol consumption (daily intake in mg/L), family history of hypertension (father and/or mother), birthweight (grams), and rapid weight gain between 2 and 4 years (change in Z score above 0.67 standard deviation) [24] . Paternal and maternal history of dyslipidemia, hypertension, myocardial infarction, and/or stroke was also considered.

The means of the variables according to the quartiles of Pep-C were evaluated according to the trend tests of the ordered groups. Due to the asymmetric distribution, C-reactive protein and triglycerides were transformed into logarithm. Multiple linear regression was used to evaluate the association between C-Peptide and cardiovascular risk factors, estimates were adjusted for confounding variables like: fasting time (estimated as the time between the last meal and blood sample collection), physical activity practice per week (minutes), family income, mother’s skin color, birthweight, rapid growth between 2 and 4 years old, smoking at 23 years, alcohol consumption, family history of dyslipidemia, arterial hypertension and stroke, myocardial infarction [23].

The analyses were carried out using the software STATA version 13.1. The study was approved by the Research Ethics Committee of the Medical School of the Federal University of Pelotas under protocol OF. 16/12. All interviews and blood collections were performed after written consent was obtained from the participants.

## Results

In 2004, 4297 individuals were interviewed, which – added to the 282 deaths identified in the cohort – represented a follow-up rate of 77.4%. In 2012, 3701 individuals were interviewed, which – given the 325 deaths identified in the cohort – represented a follow-up rate of 68.1%.

In ad-doc tests in the regression models, sex was not shown an interaction condition with the Pep-C values, because of these findings, the analyzes were not stratified between sex. The exclusion criteria used for Hs-CRP greater than 10 mg/L withdraw participants and the hyperglycemia in the casual glucose dosage (> 200 mg / dL), totaling the exclusion of 02 individuals at the 23ª and 25 participants at 30ª. Table 1 shows the distribution of the studied population according to the outcomes. C-Peptide was measured in 3807 subjects and the geometric mean was 1.6 ng/mL (CI 95% 1.5; 1.6). From 23 to 30 years, the mean total cholesterol increased from 155.9 to 190.6 mg/dL, HDL from 55.5 to 58.7 mg/dL, the geometric mean of triglycerides from 88.4 to 100.4 mg/dL, and Hs-CRP from 2.0 to 1.47 mg/dL. Concerning the anthropometric measures, mean waist circumference increased from 78.2 to 84.7 cm and BMI from 23.6 to 26.8 kg/m^2^. Systolic blood pressure increased from 117.4 to 121.0 mmHg and diastolic from 73.5 to 70.7 mmHg.

**Table 1:** Participant characteristics at 23 and 30 years old

Variables	23 years		30 years	
	n	μ (CI95%)	n	μ (CI95%)
Anthropometric				
Weight (kg)	4263	67.0 (66.6; 67.46)	3525	75.6 (75.0; 76.2)
Height (cm)	4263	167.4 (167.1; 167.7)	3581	167.7 (167.4; 168.0)
Waist circumference (cm)	4260	78.2 (77.9; 78.5)	3540	84.7 (84.3; 85.1)
Body mass index	4136	23.6 (23.4; 23.7)	3508	26.8 (26.6; 27.0)
Metabolic				
Serum glycaemia (mg/dL)	3713	97.1 (96.6; 97.6)	3506	87.9 (87.4; 88.4)
Total cholesterol (mg/dL)	2417	155.9 (154.4; 157.3)	3506	190.6 (189.4; 191.8)
HDL cholesterol (mg/dL)	3801	55.5 (55.0; 55.9)	3506	58.7 (58.2; 59.1)
LDL cholesterol (mg/dL)			3506	109.4 (108.4; 118.1)
Triglycerides ^a^ (mg/dL)	3801	88.4 (86.9; 89.9)	3506	100.4 (98.5; 102.2)
C-reactive protein (mg/dL) #	3449	2.0 (2.0; 2.1)	3188	1.47 (1.4; 1.5)
Glycated hemoglobin (mg/dL)			3516	51.1 (5.0; 5.1)
C-Peptide ^a^ (ng/mL)	3807	1.6 (1.5; 1.6)		
Hemodynamic				
Systolic arterial pressure (mmHg)	4291	117.4 (116.9; 117.9)	3593	121.0 (120.6; 121.5)
Diastolic arterial pressure (mmHg)	4291	73.5 (73.2; 73.9)	3593	75,3 (75.0; 75.6)
Heart rate (bpm)	4291	74.5 (74.1; 74.8)	1565	70.7 (70.1; 71.3)
Behavioral				
Prevalence of sedentary lifestyle	4269	444.8 (428.3; 461.2)	3580	294.7 (282.5; 307.0)

μ: arithmetic mean;# ^a^: Geometric mean

At 23 years, in transversal analysis, C-Peptide was positively associated with waist circumference, BMI, glycaemia, total cholesterol, triglycerides, and Hs-CRP, whereas a negative association was observed with HDL. Longitudinally at 30 years, C-Peptide remained associated with waist circumference and BMI at 30. Concerning the metabolic risk factors, only triglycerides and Hs-CRP at 30 years were associated with C-Peptide. At 23 and 30 years, only diastolic blood pressure was associated with C-Peptide, but this association did not show a linear pattern (Table 2).

**Table 2:** Cardiovascular risk factors according to C-Peptide quartiles at 23 and 30 years old

Cardiovascular risk factors	C-Peptide				^b^P Trend value
	1st quartile	2nd quartile	3rd quartile	4th quartile	
	23 years				
	*n* = 968	*n* = 958	*n* = 1015	*n* = 865	
Anthropometric					
Waist circumference (cm)	76.6 (75.9; 91.0)	77.3 (76.7; 77.9)	78.3 (77.6; 78.9)	80.8 (79.9; 81.6)	**< 0.001**
Body mass index	22.7 (22.5; 22.9)	23,4 (23.1; 23.6)	23.7 (23.4; 23.9)	24.7 (24.3; 25.1)	**< 0.001**
Metabolic					
Capillary glycaemia (mg/dL)	92.1 (91.4; 92.9)	94.4 (93.6; 95.2)	97, 9 (96.9; 98.8)	104.8 (103.7; 106.0)	**< 0.001**
Total cholesterol (mg/dL)	155.0 (152.2; 157.9)	154.5 (151.9; 157.2)	156.3 (153.4; 159.2)	157.9 (154.9; 161.0)	0.094
HDL cholesterol (mg/dL)	56,2 (55.3; 57.0)	55.6 (54.8; 56.4)	55.2 (54.4; 56.0)	54.9 (54.1; 55.8)	**0.032**
Triglycerides (mg/dL)^a^	76,5 (74,1; 78,9)	76,5 (74,1; 90,7)	96,3 (93,2; 99,5)	108,4 (104,4; 112,6)	**< 0.001**
C-reactive protein (mg/dL)^a^	1,0 (1,06; 1,08)	1,0 (1,06; 1,17)	1,3 (1,20; 1,39)	1,4 (1.3; 1.5)	**< 0.001**
Hemodynamic					
Systolic arterial pressure (mmHg)	118.2 (117.2; 119.2)	116.7 (115.8; 117.6)	116.7 (115.7; 117.6)	117.5 (116.6; 118.6)	0.356
Diastolic arterial pressure (mmHg)	73.9 (73.2; 74.7)	72.6 (71.9; 73.3)	72,9 (72.2; 73.6)	73.9 (73.1; 74.6)	0.765
Heart rate (bpm)	73.0 (72.2; 74.0)	73.3 (72.6; 74.0)	75.9 (75.2; 76.7)	76.30 (75.4; 77.0)	**< 0.001**
	30 years				
	*n* = 966	*n* = 955	*n* = 1006	*n* = 857	
Anthropometric					
Waist circumference (cm)	83.0 (82.1; 83.7)	84.2 (83.4; 85.1)	85.0 (84.1; 86.0)	87.7 (86.6; 88.8)	**< 0.001**
Body mass index	25.8 (25.4; 26.1)	26.6 (26.2; 27.0)	27.1 (26.7; 27.5)	28.3 (27.8; 28.8)	**< 0.001**
Metabolic					
Serum glycaemia (mg/dL)	88.3 (87.2; 89.4)	87.3 (86.2; 88.3)	87.9 (86.8; 88.9)	88.5 (87.2; 89.8)	0.702
Total cholesterol (mg/dL)	189.9 (187.3; 192.6)	190.7 (188.1; 193.3)	191.0 (188.4; 193.5)	192.1 (189.3; 194.9)	0.084
HDL cholesterol (mg/dL)	59.1 (58.1; 60.0)	58.8 (57.8; 59.7)	58.5 (57.5; 59.4)	58.6 (57.5; 59.7)	0.223
LDL cholesterol (mg/dL)	109.4 (107.3; 111.6)	109.3 (107.3; 111.4)	109.7 (107.6; 111.8)	110.3 (108.1; 112.4)	0.161
Triglycerides (mg/dL)^a^	94.5 (91.0; 98.1)	100.5 (96.6; 104.6)	101.9 (97.9; 105.9)	105.4 (101.0; 111.0)	**< 0.001**
C-reactive protein (mg/dL)^a^	1.17 (1.07; 1.29)	1.43 (1.31; 1.57)	1.64 (1.50; 1.79)	1.83 (1.66; 2.00)	**< 0.001**
Glycated hemoglobin (%)	5.06 (5.03; 5.09)	5.10 (5.07; 5.14)	5.11 (5.08; 5.15)	5.10 (5.06; 5.14)	0.087
Hemodynamic					
Systolic arterial pressure (mmHg)	122.0 (121.0; 122.8)	120.4 (119.5; 121.4)	121.7 (120.0; 121.6)	121.1 (120.0; 122.2)	0.215
Diastolic arterial pressure (mmHg)	75.1 (74.17; 75.8)	74.8 (74.2; 76.1)	75.4 (74.8; 76.0)	76.4 (75.6; 77.14)	**0.003**
Heart rate (bpm)	69.7 (68.5; 70.9)	70.5 (69.3; 71.7)	72.1 (69.8; 72.5)	71.2 (70.0; 72.5)	**0.014**

^a^μ Geometric mean (CI95%)

^b^Test for trend across ordered groups

Values presented in bold present statistical significance

Considering that the independent variable of positive family history for myocardial infarction was not significant in any of the multivariate stages used in the analysis (p = 0.56), it was thus excluded from participation in subsequent level adjustments. Table 3 shows that, even after adjusting for confounding factors, C-Peptide remained associated with waist circumference and BMI at 23 and 30 years of age. Among the metabolic variables at 23 years, C-Peptide was positively associated with glycaemia, triglycerides, and Hs-CRP and negatively with HDL cholesterol. In the longitudinal analysis at 30 years, C-Peptide was positively associated with glycated hemoglobin, triglycerides, and HS-CRP, and negatively with HDL cholesterol. Regarding hemodynamic variables, C-Peptide was not associated with systolic blood pressure, whereas diastolic blood pressure at 30 years was higher among those subjects in the upper quartile of C-Peptide.

**Table 3:** Multivariate regression of cardiovascular risk factors and C-Peptide at 23 and 30 years old^a^

Cardiovascular risk factors	1st quartile	2nd quartile	3rd quartile	4th quartile
	μ (CI95%)	β (CI95%)	β (CI95%)	β (CI95%)
	23 years			
Anthropometric				
Waist circumference (cm)	Reference	0.88 (− 0.46; 2.22)	**1.78 (0.41; 3.15)**	**4.90 (3.45; 6.36)**
	Reference	0.49 (−0.38; 1.02)	**0.97 (0.44; 1.52)**	**2.15 (1.57; 2.72)**
Metabolic				
Serum glycaemia (mg/dL)	Reference	**2.58 (0.67; 4.50)**	**5.55 (3.60; 7.49)**	**11.19 (9.80; 13.94)**
Total cholesterol (mg/dL)	Reference	−0.38 (−5.04; 4.28)	0.95 (−3.79; 5.70)	1.76 (− 3.28; 6.80)
HDL cholesterol (mg/dL)	Reference	**−2.12 (−3.80; − 0.44)**	**− 2.33 (− 4.04; − 0.61)**	**− 2.10 (− 3.93; − 0.29)**
Triglycerides (mg/dL)	Reference	**1.12 (1.05; 1.20)**	**1.19 (1.05; 1.20)**	**1.28 (1.19; 1.38)**
C-reactive protein (mg/dL)	Reference	**0.94 (0.81; 1.10)**	**1.26 (1.08; 1.47)**	**1.42 (1.20; 1.67)**
Hemodynamic				
Systolic pressure (mmHg)	Reference	−1.32 (−3.39; 0.75)	− 1.93 (−4.05; 0.18)	−0.25 (−2.48; 2.01)
Diastolic pressure (mmHg)	Reference	**−1.88 (− 3.43; − 0.32)**	**−1.93 (− 3.52; − 0.35)**	−0.20 (− 1.88; 1.47)
	30 years			
Anthropometric				
Waist circumference (cm)	Reference	1.0 (−0.39; 2.41)	**1,74 (0.30; 3.17)**	**4.18 (2.65; 5.70)**
Body mass index	Reference	**0.75 (0.15; 1.34)**	**1.26 (0.65; 1.88)**	**2.26 (1.61; 2.91)**
Metabolic				
Serum glycaemia (mg/dL)	Reference	−1.28 (−3.18; 0.62)	−0.39 (−2.33; 1.55)	−0.66 (− 2.72; 1.40)
Glycated hemoglobin (%)	Reference	**0.06 (0.01; 0.12)**	**0.07 (0.02; 0.13)**	**0.07 (0.01; 0.14)**
Total cholesterol (mg/dL)	Reference	−0.62 (−4.93; 3.68)	−0.69 (−5.11; 3.71)	−2.20 (−6.88; 2.47)
HDL cholesterol (mg/dL)	Reference	−0.79 (−2.42; 0.84)	−1.30 (− 2.97; 0.36)	**−1.82 (− 3.59; − 0.05)**
LDL cholesterol (mg/dL)	Reference	−0.76 (− 4.23; 2.70)	−0.68 (− 4.23; 2.86)	−0.98 (− 4.75; 2.78)
Triglycerides (mg/dL)	Reference	**1,06 (0,99; 1,13)**	**1,07 (1,0; 1,14)**	**1,06 (0,99; 1,14)**
C-reactive protein (mg/dL)	Reference	**1,23 (1,06; 1,42)**	**1,51 (1,30; 1,75)**	**1,54 (1,32; 1,80)**
Hemodynamic				
Systolic pressure (mmHg)	Reference	−0.96 (−2.54; 1.62)	−0.25 (−1.87; 1.36)	−0.03 (− 1.75; 1.68)
Diastolic pressure (mmHg)	Reference	−0.44 (−1.53; 0.65)	0.45 (− 0.66; 1.56)	1.12 (− 0.06; 2.30)

μ geometric mean

^a^adjusted for: time of fasting, physical activity practice per week (minutes), family income, mother’s skin color, birthweight, rapid growth between 2 and 4 years old, smoking at 23 years, alcohol consumption, family history of dyslipidemia, arterial hypertension and stroke, myocardial infarction

Values presented in bold present statistical significance

## Discussion

In the present study, the cross-sectional analysis at 23 years showed that C-Peptide was positively associated with waist circumference, BMI, glycaemia, triglycerides, and Creactive protein, whereas the association with HDL cholesterol was in the opposite direction. In the longitudinal analysis, C-Peptide remained positively associated with waist circumference, BMI, glycated hemoglobin, triglycerides, and C-reactive protein and negatively with HDL cholesterol. The direction of the association with diastolic blood pressure changed, i.e., at 23 years’ diastolic blood pressure was higher among those in the first quartile of C-Peptide whereas the opposite was observed at 30 years.

Studies on the effect of C-Peptide on glycaemia have yielded conflicting evidence. Whereas Hoogwerf et al. found no association [24], Oskarsson et al. reported that C-Peptide enhances insulin-induced hypoglycemia among diabetic patients [25]. Nordquist et al. showed that C-Peptide infusion in patients with type-I diabetes improves glucose use by about 25% [26]. In the present study, C-Peptide was positively associated with glycaemia at 23 years, but no association was observed in the longitudinal analysis at 30 years. That may be due to the increase in insulin resistance, which would be the result of the rise in the prevalence of overweight – from 28.3 to 57.6% – from 23 to 30 years [27], increasing the peripheral resistance to insulin, which, along with hyperinsulinemia, would result in a temporary reduction in glycemic status.

On the other hand, the findings of the associations with abdominal circumference and body mass index are based on the understanding that increased visceral fat contributes to insulin resistance and pro-inflammatory adipokine production by means of monocyte attraction protein (MCP-1) in addition to its greater performance on catecholaminergic receptors and lower performance on insulin receptors, which favors a greater release of free fatty acids and hypertriglyceridemia, which in turn favors the lipotoxicity phenomenon, which was transversely identified and remained associated longitudinally at 30 years [28, 29].

C-Peptide was associated with triglycerides at 23 and 30 years. Although they cannot be considered a marker of atherogenic risk by themselves, triglycerides in association with other components such as obesity, diabetes, or hypertension are a marker of cardiovascular risk [30]. In addition to this condition, the negative relation with HDL cholesterol at 23 and 30 years old stands out. Suggesting that C-Peptide is associated with metabolic factors related to cardiovascular risk. C-reactive protein, a marker of systemic inflammatory process, was considered a cardiovascular risk factor [31–34]. Danesh (2004), in a meta-analysis of observational studies, observed that those in the highest tercile of C-reactive protein were at higher risk of cardiovascular events (OR 1.45; CI95% 1.25–1.68) [35]. However, a Mendelian randomization study showed that Hs-CRP was not independently associated with cardiovascular risk and that it could be a marker of risk [36]. In the present study, C-Peptide remained associated with Hs-CRP at 23 and 30 years old, which suggests that C-Peptide is associated with the systemic inflammatory process.

Diastolic blood pressure was positively associated with C-Peptide at 23 years, but, unlike Chen and Every [37, 38] systolic blood pressure was not associated with C-Peptide. Although it accepts the association between insulin resistance and the condition of hypertension due to favoring the conditions of vasoconstriction, greater renal resorption of sodium and increased production of angiotensin II at the level of visceral fat. Which in turn, leads to less production of oxide nitric acid and hypertrophy in the mucosal mean of vessels by the action of hyperinsulinemia, our findings did not observe association with the tensional state and the levels of the pep-C quartiles at 23 or 30 years.

The majority prevalence of diabetes in developing countries, stay in the 45 to 64 -year age range, in contrast, with developed countries (> 64 years of age) [39]. Our findings are in line with the epidemiological profile indicated, since the associates found to be pre-findings point to a condition of insulin resistance associated with conditions of cardiometabolic risk already present at 23^a^ and is maintained longitudinally at the end of the 3^a^ decade of life. A strength of this study is its population-based design and the large proportion of the sample successfully followed, which minimized the likelihood of selection bias. Among the limitations, it could be pointed that the biochemical tests were carried out in non-fasting samples. On the other hand, more recent evidence indicates that casual exams allow better risk estimate [40] and, furthermore, the analyses were adjusted for time of fasting.

## Conclusion

**Fig. 1:**
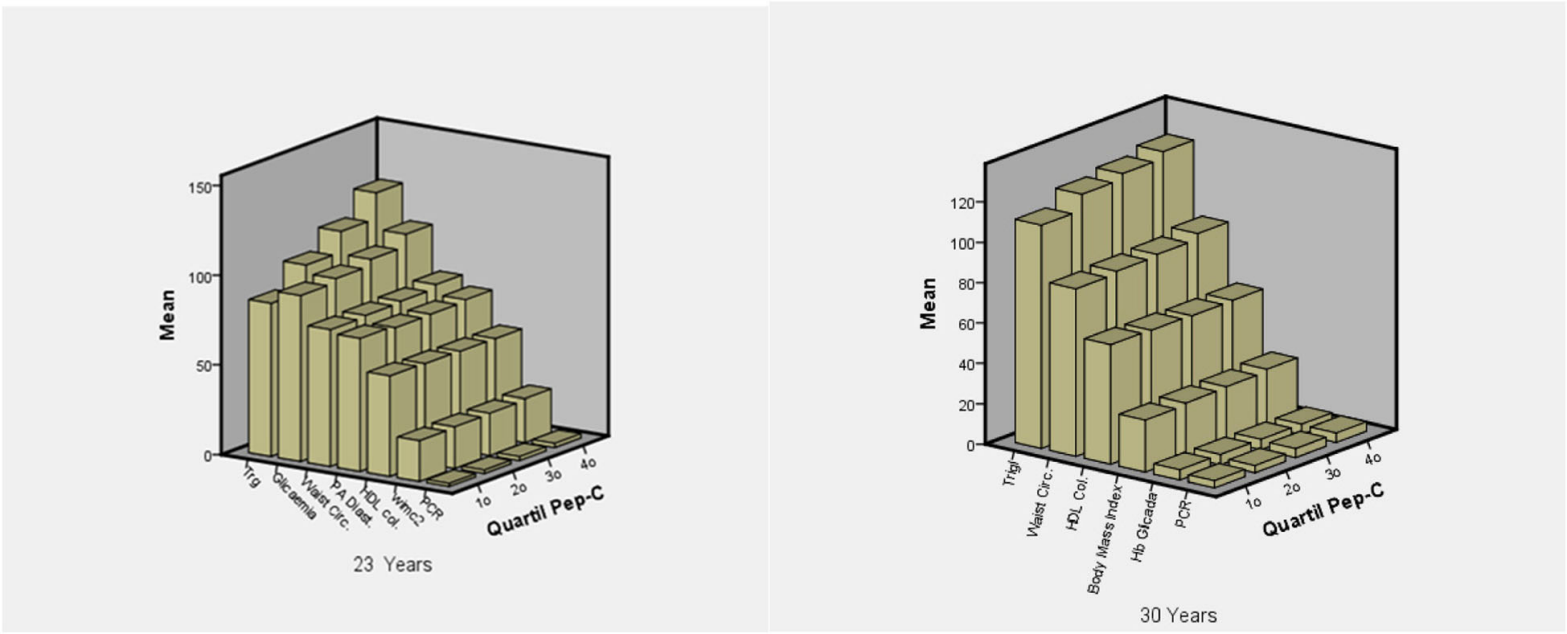
Cardiovascular risk components associated with Pep-C at 23 and 30 years

In a population that has been prospectively followed since birth, we observed a positive relationship between C-Peptide and obesity indicators such as waist circumference and BMI at 23 years and 30 years of age (as shown in Fig. 1). C-Peptide was also associated with the systemic inflammatory process and the higher level of metabolic cardiovascular risk factors.

## Abbreviations

23^a^: Twenty-three years
30^a^: Thirty years
BMI: Body mass index
cm: Centimeters
C-Peptide: Insulin-binding peptide
HbA_1c_: Glycated hemoglobin
*HDL*: High-density lipoprotein **–** HDL cholesterol
HR: Hazard ratio
HS-CRP: High-sensitivity C-reactive protein
IPAQ: International physical activity questionnaire
kg: Kilograms
LDL: Low-density lipoprotein – LDL cholesterol
MCP-1: Monocyte attraction protein
mg/dL: Milligrams per deciliter
mm: Millimeters
mmHg: Millimeters of mercury
ng/mL: Nanogram per milliliter
RR: Relative risk
tPA: Tissue plasminogen activator
μ: Geometric mean
